# Task and Participant Scheduling of Trading Platforms in Vehicular Participatory Sensing Networks

**DOI:** 10.3390/s16122013

**Published:** 2016-11-28

**Authors:** Heyuan Shi, Xiaoyu Song, Ming Gu, Jiaguang Sun

**Affiliations:** 1 School of Software, Tsinghua University, Beijing 100084, China; guming@tsinghua.edu.cn (M.G.); sunjg@tsinghua.edu.cn (J.S.); 2 Department of Electrical & Computer Engineering, Portland State University, P.O. Box 751, Portland, OR 97207-0751, USA; song@ece.pdx.edu

**Keywords:** participatory sensing networks, vehicular sensor networks, tasks selection, participants recruitment, scheduling

## Abstract

The vehicular participatory sensing network (VPSN) is now becoming more and more prevalent, and additionally has shown its great potential in various applications. A general VPSN consists of many tasks from task, publishers, trading platforms and a crowd of participants. Some literature treats publishers and the trading platform as a whole, which is impractical since they are two independent economic entities with respective purposes. For a trading platform in markets, its purpose is to maximize the profit by selecting tasks and recruiting participants who satisfy the requirements of accepted tasks, rather than to improve the quality of each task. This scheduling problem for a trading platform consists of two parts: which tasks should be selected and which participants to be recruited? In this paper, we investigate the scheduling problem in vehicular participatory sensing with the predictable mobility of each vehicle. A genetic-based trading scheduling algorithm (GTSA) is proposed to solve the scheduling problem. Experiments with a realistic dataset of taxi trajectories demonstrate that GTSA algorithm is efficient for trading platforms to gain considerable profit in VPSN.

## 1. Introduction

Participatory sensing is an attractive paradigm in cyber physical systems and has shown its great potential in various applications. It was originally proposed recruiting ordinary citizens to collect and share massive amounts of sensory data using their portable smart devices [1]. Vehicular participatory sensing networks (VPSNs) is a typical participatory sensing architecture in vehicular sensor networks (VSN). VSN is a promising paradigm for monitoring the physical world when nowadays there are numerous vehicles equipped with sensing, communication, and computation devices [2]. In VSN, probe vehicles, e.g., taxicabs, buses, private cars and special vehicles, continuously gather, process, and share location relevant sensor data [3]. The vehicles on the road act as the owner of mobile sensors to monitor the physical world by its sensors and other smart devices on vehicles.

**Figure 1:**
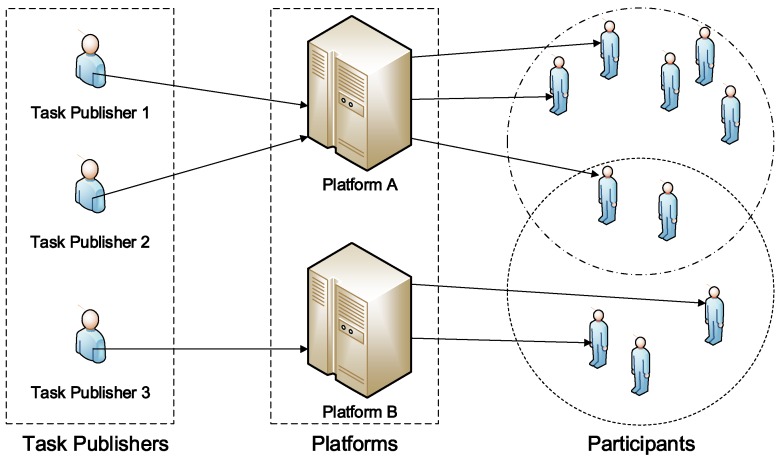
The participatory sensing system with multiple trading platforms, where the task publishers can choose different platforms for their sensing tasks.

There are three categories of the main stakeholders in a general participatory sensing system: task publishers, trading platforms, and participants [1], which is showed in Figure 1. A task publisher is the provider of tasks. When a certain type of sensory data is mandatory, a task publisher publishes corresponding sensing tasks with the detailed requirements and payment. The requirements of the task contain spatial-temporal requirements and sampling amounts which depend on the application. The payment is the reward to the platform for the participant’s recruitment. A trading platform in vehicular participatory networks is a medium between tasks and participants, by receiving task requirements from publishers and recruiting participants in terms of accepted tasks’ requirements. A trading platform is operated by the payment from publishers and recruit participants on behalf of publishers. It keeps publishers from recruiting participants directly and makes participants access sensing tasks easily. Participants are candidates who wait for recruitment by a trading platform. A participant provides a bid and a predictable trajectory with the sensing ability to trading platforms.

Some literature confuses the publisher with the trading platform, ignoring the incentive of the trading platforms. Only task publishers and participants are considered, without a specific trading platform [4]. In this case, a trading platform is supposed to be part of one or more task publishers. The publishers play the role of the trading platform to recruit participants, without considering the profit of the trading platform. The purpose of a trading platform is to maximize the quality of sensing data under the budget constraints, which are the same as task publishers, rather than to maximize the profit of its own.

However, in practice, task publishers and trading platforms should be treated separately, since they are two different independent economic entities [1]. Moreover, it is impractical for a single publisher to set up a perfect trading platform, which is able to recruit enough participants and obtain high-quality data in VPSN. And the low utilization, as well as the high costs of the developments and maintenance, is an issue for a trading platform if it is dedicated to the task publisher.

Therefore, we assume that a practical trading platform in markets of VPSN is settled by a third party. In this case, the trading platform is an independent member in VPSN. And how to maximize the profit gained from trading is the main issue for a trading platform in VPSN.

**Figure 2:**
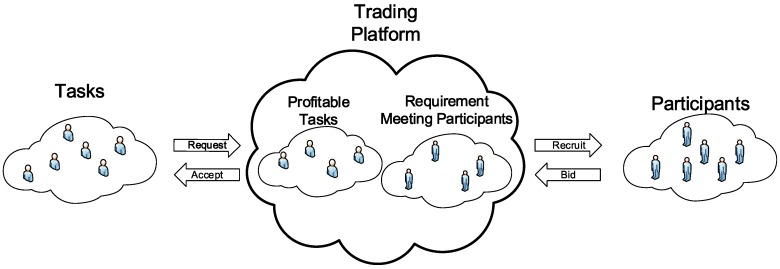
The process of a trading platform in VPSN.

Figure 2 shows the process of a trading platform. When receiving a task request, the trading platform decides whether to accept the task or not, in terms of the profit that this task will bring. It is noted that each component of the system may not be single, as shown in Figure 1. A task publisher may send tasks to multiple trading platforms for a better sensing quality or price. So trading platforms in the system face competition with each other. Therefore, we suppose that a trading platform accepts a task only if it does not suffer a loss of profit. If the payment of this task is considerable for a trading platform, the platform will accept the task and select suitable participants with fewer costs to meet the requirements of the task.

For a trading platform in VPSN, an optimization problem can be formulated to maximize the profit by making a scheduling, including tasks selection and participants recruitment. A trading platform selects tasks with profitable payment and minimizes the sensing costs of satisfying data requirements of each accepted task. By different scheduling for selection of tasks and recruitment of participants, the trading platform gains a varying amount of profit.

This paper discusses the scheduling problem of the trading platforms in VPSN. We seek to maximize the profit of a trading platform, under the considerations of payment from publishers and costs from participants. In VPSN, vehicles have the distinct advantage of predictable mobility, which brings new insight into improving the crowdsourcing quality [5]. So predictable mobility of vehicles is adopted when designing a schedule of trading platforms to make profits.

The contributions of this paper can be summarized as follows.

- We formally give a system model of participatory sensing in vehicular sensor networks for the scheduling of trading platform.
- A Genetic-based Trading Scheduling Algorithm (GTSA) is proposed to solve the scheduling problem of trading platforms under participants’ predictable trajectories and task requirements.
- The simulation based on realistic traffic trajectories of taxi proves that the GTSA significantly enhances the profit of trading platform.

The rest of this paper is organized as follows. We first review the related work on the tasks selection and participants recruitment with different constraints in Section 2. In addition, the system model of VPSN, as well as the scheduling problem formulation, is given in Section 3. Then the GTSA algorithm is proposed in Section 4. The simulation with realistic trajectories of taxis in Beijing is presented in Section 5. Finally, we discuss the future works in Section 6 and conclude this paper in Section 7.

## 2. Related Work

The problem that maximizes the profit of trading platform in VPSN has not been specifically considered in the literature. However, the scheduling of selecting tasks and/or participants to achieve different goals has been considered by many researchers, under various constraints.

The coverage of spatial sensing is the principal goal of sensing tasks. Many schemes and algorithms are adopted to maximize the spatial coverage of tasks under the constraint of energy, in various types of sensing network. In urban sensing, Khan et al. propose a novel framework called PLUS for data collection, and an energy efficient localization scheme called sLoc in participatory sensing [6]. The goal of this is to ensure coverage requirements with energy constraints. Weinschrott et al. propose a coverage metric for assessing the completeness of sensing that considers spatial and temporal aspects [7]. To maximize coverage while minimizing energy consumption of mobile nodes, a centralized and a distributed coordination algorithm is presented to select sensing nodes. Bazzi et al. adopt radio access technologies instead of the cellular network in urban scenarios to offload the cellular resources [8]. For wireless sensing networks, Dong et al. propose a novel event data collection approach named reliability and multipath encounter routing (RMER) for meeting reliability and energy efficiency requirements [9]. By RMER, fewer monitor nodes are selected in hotspot areas that are close to the Sink, and more monitor nodes are selected in non-hotspot areas. Liu et al. propose an active detection-based security and trust routing scheme named ActiveTrust [10]. ActiveTrust scheme determines routes with fully using of energy in non-hotspots to create as many detection routes as needed to achieve the desired security and energy efficiency. For wireless software defined networks (WSDNs), a fast program codes dissemination (FPCD) scheme for smart wireless software defined networking is proposed [11]. FPCD scheme aims to save energy of spreading program codes by selecting many nodes in the area far from the sink but less number of active nodes near the sink.

Some researchers consider the participant recruitment with constraints of quality-of-information (QoI), which relates to judge whether the information fits for particular purposes [12]. When a certain type of sensory data is required, the task publisher publishes a corresponding sensing tasks with a detailed QoI and the amount of affordable budget to be paid to the participants [3]. Based on the constraints of QoI, [13] identifies the well-suited participants for data collection, in terms of geographic and temporal availability as well as participation habits. [14] considers the quality of sensed data and the dedicated energy for their acquisition under the QoI and energy-aware mobile sensing scheme. The subset of users, similar to participants in VPSN, is selected based on the tabu search, a meta-heuristic algorithm, to maximize the amount of non-redundant information while minimizing the overall energy consumption. [15] deals with the issues related to resource constraints, user privacy, data reliability, and uncontrolled mobility. In addition, [16] aims to optimize deployment of data collection points. It provides an efficient recruitment strategy of participants in high-quality sensing and reliable sensing data collection and communication. However, these papers focus on the participant recruitment without considering the tasks selection. In other words, the single or multiple tasks should be accepted and completed with no consideration on whether the payment of a task is acceptable to recruit adequate participants.

The constraints of the budget are to be incorporated into the participant selection. Based on the budget constraints, a publisher can be allowed to only select part of the candidate participants to collect data based on the constrained budget. The problem often appears to be a multi-objective optimization (MOO) problem. [4] converts QoI requirements of multiple concurrent tasks with different budget constraints to a nonlinear knapsack problem. A dynamic participant selection (DPS) strategy is adopted in order to maximize QoI under the budget constraints. [17] studies the participant selection in two conditions, the fixed budget constrained for every time stamp and dynamic budget constrained for the entire campaign, respectively. Several heuristics for the online version are proposed, which exploit the spatial and temporal knowledge acquired over time. [18] considers the participant’s reputation and try to maximize total data credibility under budget constraint for participatory sensing systems. A maximum sensing data credibility problem is defined then a trustable participants selection algorithm is presented to obtain a near optimal solution. [19] proposes an event-driven QoI-aware participatory sensing framework with energy and budget constraints and a two-step heuristic solution is proposed where the coarse-grained detection step finds its approximation and the fine-grained detection step identifies the exact location. However, to satisfy the constraints of the budget, these papers still only focus on selecting participants rather than tasks. Moreover, the goal in this case is to maximize the requirements under the budget constraints. The assumption is that a task publisher expects to maximize the gain under the payment.

Besides the participant’s recruitment on the side of tasks publishers, the tasks selection is also considered. [20] proposes the SWDCP-SCmules data collection framework to collect data generated by intelligent devices and forward them to data centers. In this framework, “SCmules” are data transmitters picking up data from nearby intelligent devices and then store-carry-forwarding them to nearby data centers, which need to weigh the value of data and to discard some less valuable data to discard when necessary. The author introduces the concept of priority to the SWDCP-SCmules scheme and gives the SA-PA algorithm to guide its priority assignment. However, the paper considers the tasks selection on the side of participants, rather than the trading platform.

## 3. Problem Formulation

In this section, a formal model for trading platform scheduling is presented firstly. In this model, the components in VPSN, tasks, participants, and the trading platform are presented. Then we formulate the scheduling problem in VPSN.

### 3.1. System Model

As discussed above, the VPSN consists of three kinds of members, task, participant and trading platform. A set of *a* tasks $\Gamma =\{\tau_{1},\cdots ,\tau_{a}\}$ with requirements and payments provided by various publishers. A set of *b* participants $P=\{p_{1},\cdots ,p_{b}\}$ with trajectories and bids. A trading platform E which selects tasks from given tasks Γ and recruits participants in candidate participants P. All the requirements of tasks and participants trajectory are in the spatial domain of $V=\{v_{1},\cdots ,v_{m}\}$ and the temporal domain of $T=\{t_{1},\cdots ,t_{n}\}$. Next, we consider each component in the VPSN respectively.

#### 3.1.1. Task Model

A task $\tau_{i}\in \Gamma$ is denoted by a tuple $\left\{R_{i},\Phi_{i}\right\}$. $R_{i}$ is a requirements matrix of temporal and spatial requirements with the sampling requirements. And $\Phi_{i}$ is the payment provided by the task publisher. Payment of a task supports the trading platform to recruit participants to complete this task. The definition of the task requirement matrix is given as follows.

**Definition** **1** (Task Requirements Matrix)**.** *Task requirements $R_{i}$ is formulated as a m×n matrix:*

(1) $$R_{i}=\left(\begin{array}{cccc} r_{11}^{i} & r_{12}^{i} & \cdots & r_{1n}^{i}\\ r_{21}^{i} & r_{22}^{i} & \cdots & r_{2n}^{i}\\ \vdots & \vdots & \ddots & \vdots \\ r_{m1}^{i} & r_{m2}^{i} & \cdots & r_{mn}^{i} \end{array}\right)$$

*where each row vector is the sampling requirements of each sensing period for a region, and each column indicates the sampling requirements of all the sensing space at a specific period. Each element in the matrix $r_{jk}\in R$ is the sampling requirements for the j spatial region in k sensing period.*

#### 3.1.2. Participant Model

A participant $p_{i}\in P$ is modeled by a tuple $\left\{C_{i},B_{i}\right\}$. $C_{i}$ is a mobility matrix indicates the predictable trajectory of a participant, with the sampling ability of $p_{i}$ in the temporal and spatial domain. $B_{i}$ is the bid provided by $p_{i}$ to be recruited by a trading platform. A participant will be recruited and complete the sensing tasks only if its bid is satisfied by the trading platform. The definition of the participant sensing capacity matrix is given as follows.

**Definition** **2** (Participant Sensing Capacity Matrix)**.** *The participant sensing capacity $C_{i}$ for a participant $p_{i}$ is an m×n matrix:*

(2) $$C_{i}=\left(\begin{array}{cccc} c_{11}^{i} & c_{12}^{i} & \cdots & c_{1n}^{i}\\ c_{21}^{i} & c_{22}^{i} & \cdots & c_{2n}^{i}\\ \vdots & \vdots & \ddots & \vdots \\ c_{m1}^{i} & c_{m2}^{i} & \cdots & c_{mn}^{i} \end{array}\right)$$

*where $c_{jk}\in C_{i}$ is the number of sampling for a participant in the j spatial region at k sensing time interval. The predictable location of this participant is determined by its mobility matrix.*

#### 3.1.3. Trading Platform Model

A trading platform *E* selects tasks and recruits participants. The accepted tasks $\Gamma^{A}$ is derived by selecting a subset of Γ , which can be defined as:

(3) $$\Gamma^{A}\subseteq \Gamma \;$$

And the recruited participants $P^{R}$ is derived by recruiting a subset of P, which is defined as:

(4) $$P^{R}\subseteq P\;$$

when making a schedule, the purpose of a trading platform is to maximize profit while meeting the requirements of selected tasks. However, if a task is accepted by the trading platform, the platform has to recruit adequate participant to get adequate sensing data for meeting the requirements of this task. Now we formally give the definition of profit and the requirements met.

**Definition** **3** (Profit)**.** *For a trading platform, the performance of a scheduling is defined by the profit G. The profit for a trading platform is derived from the payment of selected tasks and the cost of recruited participants.*

(5) $$G=\Gamma^{A}.pay-P^{R}.bid$$

*where the payment of selected tasks*

(6) $$\Gamma^{A}.pay=\sum_{\tau_{i}\in \Gamma^{A}}\Phi_{i}$$

*and the cost of recruited participants*

(7) $$P^{R}.bid=\sum_{p_{j}\in P^{R}}B_{j}\;$$

For deriving the definition of requirement met degree, we first define MAX{X} to get the maximum element in a set X. Then the accepted tasks requirement and recruited participants sampling are determined as follows.

**Definition** **4** (Accepted Tasks Requirements Matrix)**.** *The requirements of accepted tasks are a matrix denoted as*

(8) $$\Gamma^{A}.req=\left(r_{jk}^{A}\right)=\left(\begin{array}{cccc} r_{11}^{A} & r_{12}^{A} & \cdots & r_{1n}^{A}\\ r_{21}^{A} & r_{22}^{A} & \cdots & r_{2n}^{A}\\ \vdots & \vdots & \ddots & \vdots \\ r_{m1}^{A} & r_{m2}^{A} & \cdots & r_{mn}^{A} \end{array}\right)$$

*For each element $r_{jk}^{A}$ in matrix $\Gamma^{A}.req$,*

(9) $$r_{jk}^{A}=MAX\left(\left\{r_{jk}^{i}|\tau_{i}\in \Gamma^{A}\right\}\right)$$

**Definition** **5** (Recruited Participants Sampling Matrix)**.** *The sampling of recruited participants is a matrix denoted as*

(10) $$P^{R}.sen=\left(\begin{array}{cccc} c_{11}^{R} & c_{12}^{R} & \cdots & c_{1n}^{R}\\ c_{21}^{R} & c_{22}^{R} & \cdots & c_{2n}^{R}\\ \vdots & \vdots & \ddots & \vdots \\ c_{m1}^{R} & c_{m2}^{R} & \cdots & c_{mn}^{R} \end{array}\right)$$

*For each element $c_{jk}^{R}$ in matrix $P^{R}.sen$,*

(11) $$c_{jk}^{R}=\sum_{p_{i}\in P^{R}}c_{jk}^{i}$$

**Definition** **6** (Requirements Met Matrix)**.** *The trading platform has to ensure that recruiting enough sampling in terms of requirements. In other words, the sum of sensing data $C^{E}$ provided by recruited participants $P^{E}$ should be larger than, or at least equal to, the total requirements $R^{E}$ of selected tasks $\Gamma^{E}$. So we define the requirements met matrix by*

(12) $$S^{M}=\left(s_{ij}\right)=P^{R}.sen-\Gamma^{A}.req=\left(\begin{array}{cccc} s_{11} & s_{12} & \cdots & s_{1n}\\ s_{21} & s_{22} & \cdots & s_{2n}\\ \vdots & \vdots & \ddots & \vdots \\ s_{m1} & s_{m2} & \cdots & s_{mn} \end{array}\right)$$

And the scheduling meets the requirements for selected tasks only if for each element $s_{ij}$ in matrix $S^{M}$,

(13) $$s_{ij}=c_{ij}^{R}-r_{ij}^{A}\geq 0$$

In other words, a scheduling meets the requirements only if all the elements at $S^{E}$ are not less than zero.

### 3.2. Problem Statement

Based on the system model, we formulate the scheduling optimization problem as follows. Given *m* spatial sensing regions $V=\{v_{1},\cdots ,v_{m}\}$, *n* temporal sensing periods $T=\{t_{1},\cdots ,t_{n}\}$, *a* tasks $\Gamma =\{\tau_{1},\cdots ,\tau_{a}\}$ with requirements $R_{i}$ and payment $\Phi_{i}$ for each task $\tau_{i}\in \Gamma$, *b* participants $P=\{p_{1},\cdots ,p_{b}\}$ with trajectory $M_{j}$ and bid $B_{j}$ for each participant $p_{j}\in P$. The trading platform selects tasks and recruits participants by a scheduling to maximize the profit. Therefore, we have

(14) $$\begin{array}{c} \max\;\;G=\sum_{\tau_{i}\in \Gamma^{A}}\Phi_{i}-\sum_{p_{j}\in P^{R}}B_{j} \end{array}$$

(15) $$\begin{array}{c} s.t.\;\left\{\begin{array}{c} s_{ij}=c_{ij}^{R}-r_{ij}^{A}\geq 0,\left(s_{ij}\right)=P^{R}.sen-\Gamma^{A}.req\\ \Gamma^{A}\subseteq \Gamma \\ P^{R}\subseteq P \end{array}\right. \end{array}$$

## 4. Genetic-Based Trading Scheduling Algorithm

In this section, a Genetic-based Trading Scheduling Algorithm (GTSA) is adopted to solve the scheduling problem for the trading platform in VPSN. GTSA is based on the genetic algorithm. The genetic algorithm is one of the meta-heuristic algorithms, which is adopted in vehicle-based network, e.g., vehicle routing and navigation [21]. We first give a motivation of the trading platform in the VPSN to illustrate the scheduling problem. And the components in GTSA, as well as the pseudo code of GTSA, are given to show the procession of GTSA. Then the typical operations are introduced which include crossover and mutation. Finally, the fitness function as the key of GTSA is discussed.

### 4.1. Motivation

**Figure 3:**
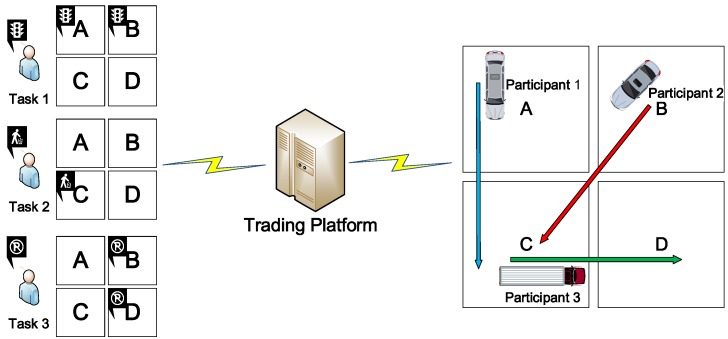
The motivation in 4 areas with 3 tasks and 3 participants.

We present the scheduling problem of the trading platform in VPSN by a motivating example, which is shown in Figure 3. There are three tasks from different publishers and three participants as the candidates. The task requirements are the spatial coverage in a set of four areas {A,B,C,D}. Due to the budget constraints, the cost of the trading platform to recruit participants can be no more than the payment of selected tasks from publishers. Otherwise the trading platform suffers the loss. Table 1 shows the requirements of tasks as well as payment. Further, Table 2 presents the bid of publishers and their trajectories.

Based on the tasks and participants presented above, the platform selects a subset of all the tasks with certain requirement and payment, according to the cost of recruiting the subset of participants. Table 3 shows various strategies of scheduling and their specific results of profit. In this simple example, the profit is maximum equals 1 when task 1 and 2 are selected. For this motivation, participants recruitment for the selected tasks is optimal with the minimal cost of recruitment by our enumeration. However, in practice, it is complex to recruit appropriate participants to minimize the cost while meeting the requirements of a task.

It should be noted that this example is quite simple that just have three tasks and participants, without considering various requirements, e.g., temporal coverage in each area, different amount of sampling of each task, and sensing ability of each participant. The practical scheme is much complex with more tasks with different requirements and participants with the different bid, trajectory and sensing ability.

### 4.2. Components Representation

The general genetic algorithm is based on the concepts of gene, chromosome, individual and population. Components in GTSA are similar to the general genetic algorithm.

**Figure 4:**
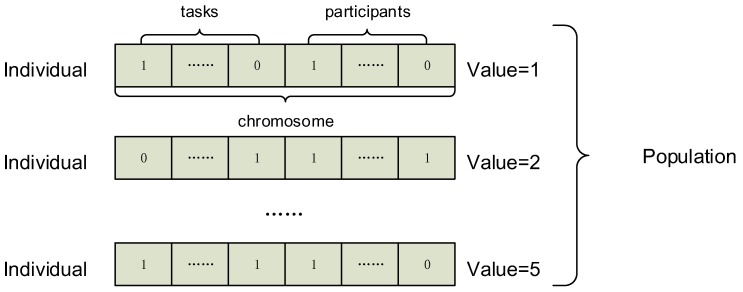
The components in GTSA.

Components in GTSA are shown in Figure 4. A population is a set of many individuals. An individual is the element of a population. Each individual consists of an array of genes called chromosome and a fitness value. The chromosome on an individual is one of the potential solutions to the scheduling problem. The fitness value is built on the chromosome and calculated by a fitness function. It depicts the degree of how this solution fits a given problem. The higher value of an individual, the better result of the solution based on this individual’s chromosome.

In GTSA, we define the chromosome for each individual as an array with binary encoding. The length of this array is the total of a number of tasks and participants. Each element of an array is a gene represents a task or a participant. The value of a gene determines that whether a task/participant is accepted/recruited by the trading platform or not. In other words, if the value of a gene is 1, the represented task/participant will be selected/recruited. Otherwise, the represented task/participant is rejected by the trading platform when its value of gene equals 0.

### 4.3. GTSA Description

We now formal give the process of GTSA. The pseudo code of GTSA is shown in Algorithm 1. The input of GTSA is the number of tasks and participants which define the length of a chromosome, as well as the maximal iteration maxIter and the population size scale which are configured by various value. The output is accepted tasks and recruited participants derived from the chromosome of individual whose value is maximal in the population after many iterations called evolution.

At the beginning of GTSA, the population is initialized at line 1. For the initialized population, the chromosome of individuals in the population is randomly generated by initializePopulation() in terms of predefined scale and given a number of tasks and participants. And the population is updated until iterate times reaching maximal iteration.

In each iteration, evolve operations including crossover() in line 3 and mutation() in line 4 is adopted. The purposes of these operations are to change the composition of a chromosome, i.e., to change the value of some genes, which may lead to a better value in the evaluation of the fitness function. The detail of operations is discussed in Section 4.4. After that, the value of each individual is derived by the fitness function fit() in line 6. The fitness function is introduced in Section 4.5. Before the end of an iteration, the new population for the next iteration is selected by selectNext() in line 8. The individual with greater value owns more possibility to keep in the population for the next evolution.

When all iterations are finished, the best individual with the most value in the population is chosen as the result of scheduling in line 10. Finally, the accepted tasks, as well as recruited participants, are returned, which is derived from the chromosome of the best individual in lines 11 and 12.

**Algorithm 1:** The pseudo code of GTSA.
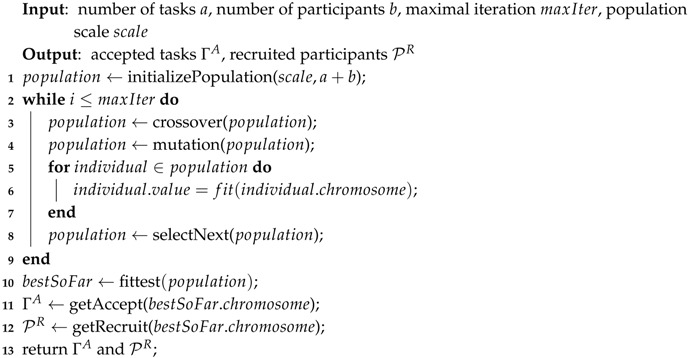


### 4.4. Evolve Operation

The main operations in GTSA are crossover and mutation, which are also the basic operations of the genetic algorithm. A changed chromosome is generated by these operations based on the previous chromosome, which may bring a greater value evaluated by the fitness function.

#### 4.4.1. Crossover

**Figure 5:**
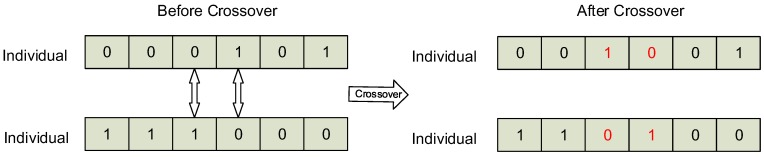
The crossover operation in GTSA.

The operation of crossover exchanges a specific part of the chromosome between two individuals. An example of crossover operation is shown in Figure 5. In this example of crossover, there are 6 genes on each chromosome. The crossover operates on the gene located at 3 and 4. The two genes, “01” and “10”, are exchanged between two chromosomes. As a result, one of the chromosomes is changed from “000101” to “001001”, the other is changed from “111000’ to “110100”.

#### 4.4.2. Mutation

**Figure 6:**
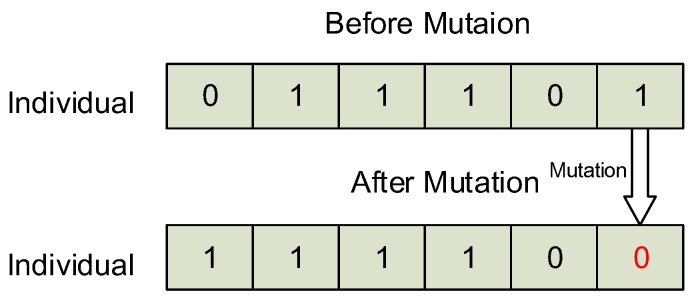
The mutation operation in GTSA.

The operation of mutation is to select a gene on a chromosome and change its value. After the operation of mutation, an individual consists of a new chromosome. And this new chromosome may synthesize a better result under the evaluation of fitness function. Figure 6 presents an example of mutation. There are 6 genes on a chromosome, the mutation operates on the last gene which changes its value from 1 to 0. Therefore the chromosome is changed from “011101” to “111100”.

### 4.5. Fitness Function

Fitness function fit(chromosome) is to evaluate the chromosome of an individual. Because the value of an individual is derived by the fitness function, and whether an individual can reserve for next evolution or not is based on the value of individual, the design of fitness function affects the performance of GTSA directly.

For the scheduling of trading platforms, the derived profit from trading is a platform care about. So it is obvious that the fitness function consists of profit (G in Equation (14)). However, the requirements of accepted tasks also have to be satisfied, which as the constraint in Equation (15). So the profit and the requirements met degree are both vital when designing the fitness function in GTSA.

To integrate requirement met degree of tasks into fitness function, we first give the definition of violation function $vio(S^{M})$

(16) $$vio\left(S^{M}\right)=\sum_{s_{ij}\in S^{M}}v_{ij}$$

where

(17) $$v_{ij}=\left\{\begin{array}{cc} 0 & s_{ij}\geq 0\\ |s_{ij}| & s_{ij}<0 \end{array}\right.$$

The violation function is the sum of absolute value of the element in the requirement met matrix. It measures the degree of a scheduling violates the accepted tasks requirements.

Based on violation function $vio(S^{M})$ and profit G, the fitness function of GTSA can be formulated as $G-vio(S^{M})$. But the value derived by the fitness function has to be positive in order to calculate the possibility of an individual to preserve for the next iteration. So we modify the initial form and give the fitness function as follows.

(18) $$fit\left(chromosome\right)=\;\left\{\begin{array}{cc} |\Gamma^{A}|-vio\left(S^{M}\right) & G<0\\ w\times G+|\Gamma^{A}|-vio\left(S^{M}\right) & G\geq 0 \end{array}\right.$$

where

(19) $$|\Gamma^{A}|=\sum_{r_{jk}^{A}\in \Gamma^{A}.req}r_{jk}^{A}$$

The factor *w* in Equation (18) is the profit weighting factor that controls the weight of profit in the fitness function. The greater value of *w* results in that the selection function tends to choose more profitable scheduling, with the increasing risk that the scheduling cannot meet the requirement of accepted tasks. The different value of the weighting factor affects the performance of GTSA in various scenarios.

In the simulation of the following section, the weight factor is 1. Moreover, it should be noted that given fitness function may not be optimal for all scenarios. In practical, the value of profit weighting factor even the form of fitness function in GTSA depends on the payment from task publishers, the bid from participants, the sample requirements and sensing ability.

## 5. Evaluation

We evaluate GTSA by the simulation based on a realistic taxi trajectories set in Beijing [22]. This is a sample of T-Drive trajectory dataset that contains one-week trajectories of 10,357 taxis. The total number of points in this dataset is about 15 million and the total distance of the trajectories reaches 9 million kilometers [23].

The GTSA is implemented by Java with two packages called JAMA and JGAP. JAMA is a fundamental linear algebra package for Java. JGAP is a Genetic Algorithms and Genetic Programming component provided as a Java framework [24]. It provides basic genetic mechanisms that can be easily used to apply evolutionary principles to problem solutions.

### 5.1. Configuration

The configuration we adopted in the simulation is as follows.

For temporal and spatial configurations, we choose the area within the Fifth Ring in Beijing and divide the area into 25 blocks. And all tasks requirements and participants trajectory are simulated in these 25 sensing blocks. The number of the sensing period we configured is 10. We assumed that each participant in VPSN reports its location per minute and repeats 10 times. In other words, the matrix of trajectory and requirements is a 25×10 matrix respectively. The maximal requirement of sampling is 10 for all the tasks in VPSN.

For configurations of tasks and participants, the number of tasks is from 100 to 1000, and the number of participants is from 100 to 1000. The requirement matrix and payment for each task are randomly synthesized. Therefore there are some tasks with low payment but high requirements, and some tasks with high payment but low requirements. The maximal payment is 10, 50 and 100 respectively while the maximal bid is always 10.

For configurations of GTSA, the population we set is 200 and the maximal iteration is 100. The threshold is 5 min so a test case is terminated if time cost over 5 min. We test each case 10 times and derive the average value of profit based on each configuration.

### 5.2. Time Cost

**Figure 7:**
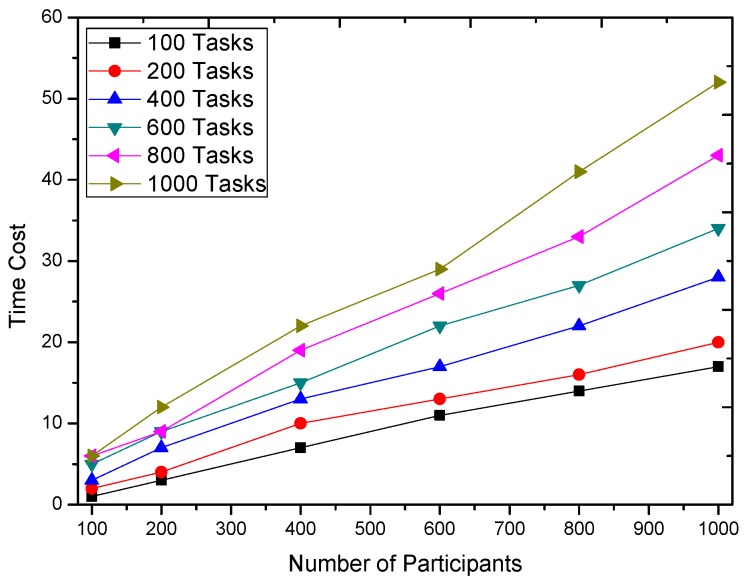
Time cost of GTSA.

We first evaluate the time cost of GTSA. The time cost of the various numbers of tasks and participants is shown in Figure 7. The time cost result demonstrates that the increasing number of tasks and participants both increase the time cost. Besides the number of tasks and participants, in fact, the size of the population and the maximal iteration times also affect the time consumption of GTSA according to the basis of the genetic algorithm.

### 5.3. Profit

To compare with GTSA, the participants offer based (POB) algorithm is adopted. POB recruits all the participants who report the trajectories, then choose the tasks that can be completed by recruited participants.

To show the profit gained by GTSA and POB clearly, the upper bound and lower bound of the profit is considered. In our simulation, the upper bound is all the payment from all the tasks. We call this scheduling as all payment scheduling. By All payment scheduling, all the tasks are accepted while no participants are recruited. It is obvious impractical because all the requirements of tasks cannot be satisfied. The lower bound is all the bids from participants. By the scheduling of all bid, the trading platform recruits all participants but accepts no tasks. In this case, the profit of trading platform losses reaches the maximal value.

It should be noticed that in practice, the lower bound is based on the scheduling that no task is selected and no participant is recruited. In this case, the profit equals 0 when the derived result less than 0. However, this bound is not used for evaluating the GTSA because it cannot properly reflect the bias between two results from GTSA and POB which are both less than 0.

**Figure 8:**
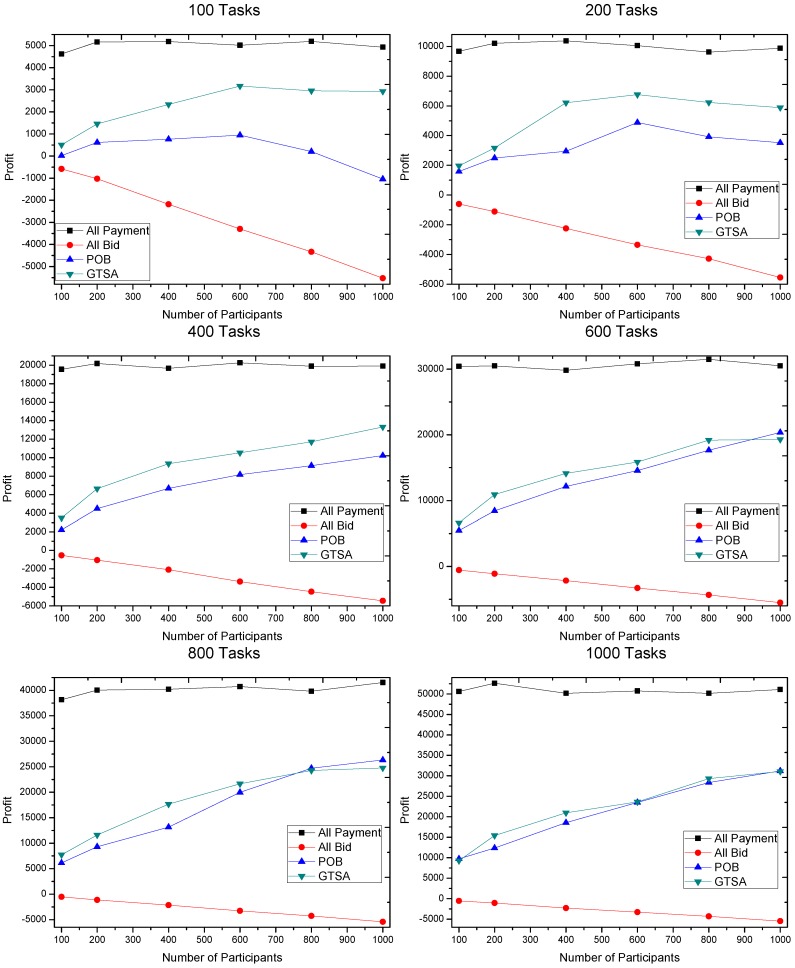
The profit gained when maximal payment of a task is 100.

**Figure 9:**
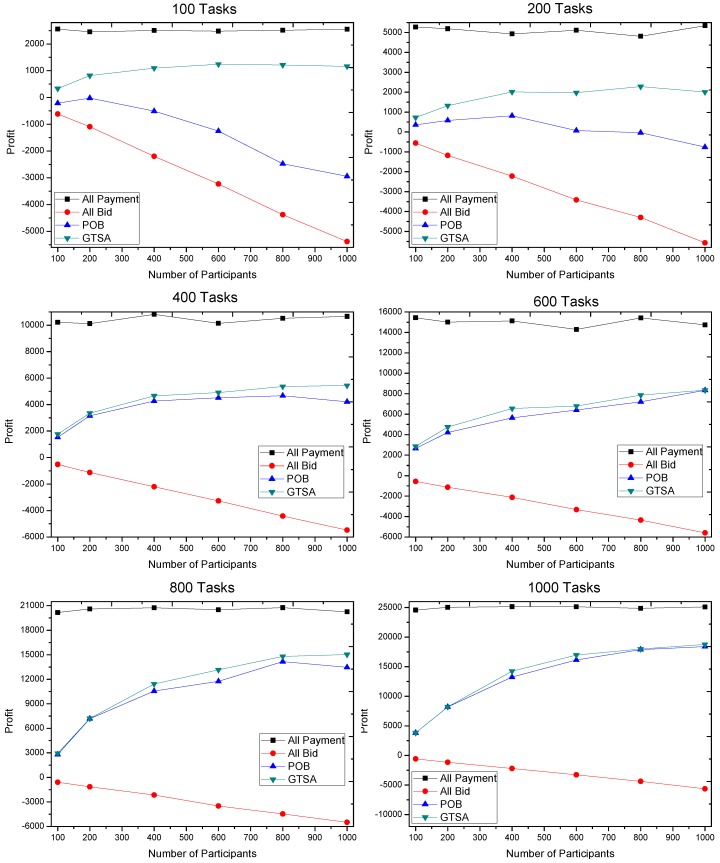
The profit gained when maximal payment of a task is 50.

**Figure 10:**
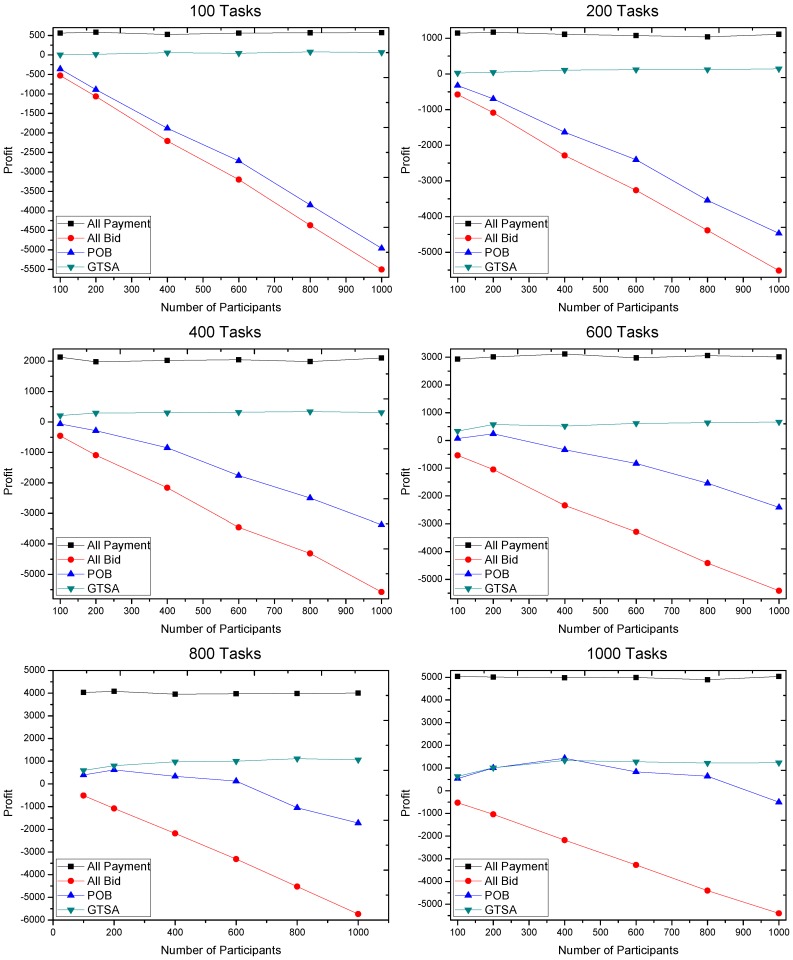
The profit gained when maximal payment of a task is 10.

The profits gained by GTSA and POB are shown in Figure 8, Figure 9 and Figure 10. The maximal payment in our simulation is configured as 100, 50 and 10 respectively, for considering different market scenarios. Based on each configuration of maximal payment, we simulate in terms that the number of tasks is 100, 200, 400, 600, 800 and 1000, and the number of participants equals 100, 200, 400, 600, 800 and 1000 respectively. Now we discuss the gained profit by two algorithms.

We first consider that the maximal payment is 50 or 100, which is relatively speaking enough for recruiting participants. The simulation results show that the profit gained by GTSA is much more than the profit by POB, when the number of tasks is relatively small, i.e., 100 and 200 tasks. However, when the number of tasks is 400 or even more, profit by GTSA and POB is comparable. It is because there are sufficient tasks with enough payment for a trading platform to recruit participants to meet the requirements of tasks.

When the payment of tasks is relatively finite for recruiting participants, i.e., 10 of maximal payment, GTSA shows its perfect performance. In this case, the average value of profit by GTSA is always more than POB significantly.

Then the changes of profit by the two algorithms when the number of participants increases is considered.

Considering GTSA with the increasing number of participants in the simulation under 50 and 100 maximal payments, when a number of tasks relative small, e.g., 100 tasks, the profit increases when the number of participants increasing at the beginning. Then the profit reaches a relatively stable value and stops to increase. It is because GTSA accepts all the profitable tasks and recruits sufficient participants with the relatively low bid. As for POB in this case, profit by POB decreases when the number of participants is relatively large. It is because that POB recruits all the participants. The trading platform still recruits participants after all the tasks accepted and all the requirements satisfied, which cause more and more unnecessary participants and cost.

When the maximal payment is 10 that is relatively restricted for trading platform, the profit increases slowly with the increasing number of participants. While profit by POB decreases in this case, even the value of profit is always negative in our simulation. The result shows that GTSA also has the great and stable performance in payment strictly limited scenarios.

### 5.4. Impact of Payment to GTSA

**Figure 11:**
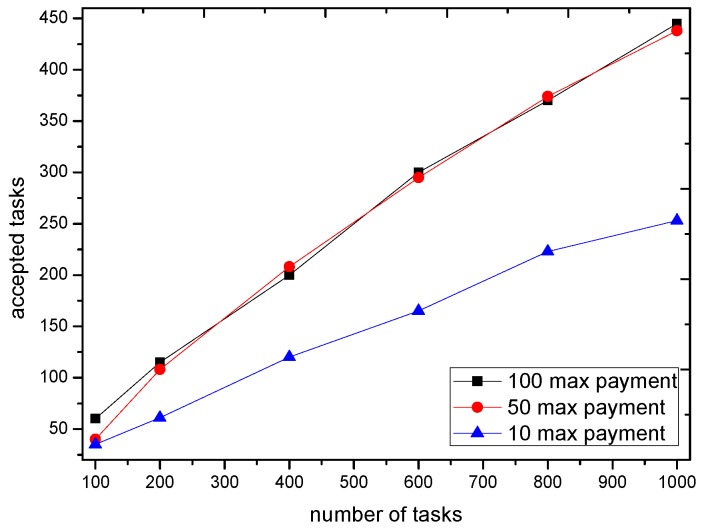
The number of accepted tasks under different maximal payment of tasks.

**Figure 12:**
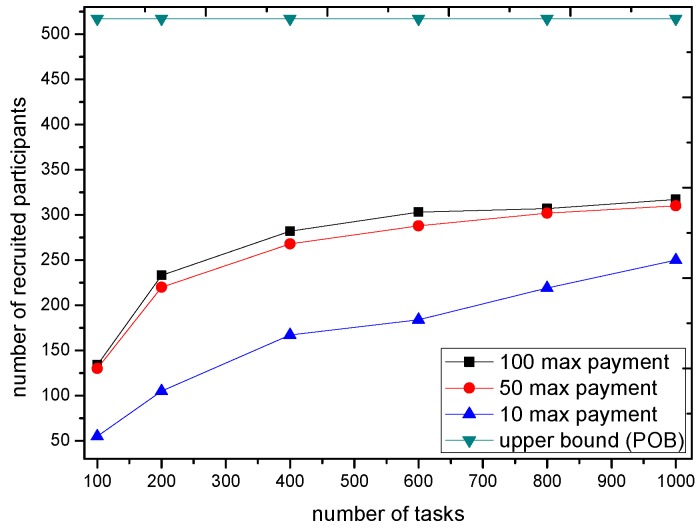
The number of recruited participants under different maximal payment of tasks.

We now consider the market impact on the GTSA. The maximal payment is configured as 10, 50 and 100 respectively to show the impact of payment when scheduling of the trading platform. The number of accepted tasks and recruited participants with different payment are shown in Figure 11 and Figure 12.

The simulation result shows that when the payment of tasks is enough for the trading platform to recruit participants e.g., 50 or 100, the number of accepted tasks as well as recruited participants is almost the same. However, with the limited payment, GTSA tend to decrease the number of accepted tasks because it cannot bring the profit by accepting tasks with relatively low payment. Moreover, the decreasing number of accepted tasks lead to the amount of requirements decreases, which reduces the number of recruited participants as the result.

### 5.5. Useless Scheduling

We discuss the correctness of GTSA. We suppose that a scheduling is a useless scheduling if the profit by this scheduling is less than 0. It is reasonable because a trading platform will stop accepting and recruiting to avoid profit losing in practice. In this case, the profit is 0.

We compare the number of useless result by GTSA and POB in different max payment of tasks of 100, 50 and 10 respectively, while the maximal bid of a participant is always 10. The number of useless scheduling are shown in Table 4. It should be noted that there are total 360 times of simulation for each kind of tasks payments.

The number of useless scheduling shows that the number of useless scheduling increases when the payment from tasks decreases. When the maximal payment of tasks is 10, which equals the maximal bid of participants, a successful scheduling is hard to find. It is because the payment of tasks is too limited to be accepted by trading platform even there are participants own the ability to complete tasks, compared to the requested bid from participants. But the validity of GTSA is also better than POB because the number of useless scheduling by GTSA is always less than POB under different payment of tasks.

## 6. Future Works

The coverage in both space and time domain is fundamental to evaluate sensing quality. Therefore, we adopt the basic sampling amount in the temporal and spatial interval to represent the requirements of tasks. Nevertheless, it is quite complex to model the requirements of tasks and evaluate the practical effects of the sensing tasks. Due to the usefulness of vehicular sensor data is considerable only if its sensing quality reaches a required level [2], the quality of this kind of tasks can only be evaluated if there are minimum data sampling collected by participants. In addition, when there are adequate samples, the improving amount of data does not affect the accuracy of the system [25]. Therefore, it is not good to collect too much information for gathering information requires payments while not improving data quality [26]. Therefore, the evaluation of tasks’ requirements should be fully considered to fit the practical scenarios.

For the simplicity of the experiments, in this paper, we suppose that the sampling requirements can be met by the sum of sampling from recruited participants, who meet the spatial and temporal constraints of tasks. And we suppose that all the participants report their predictable trajectories truly and accurately.

**Figure 13:**
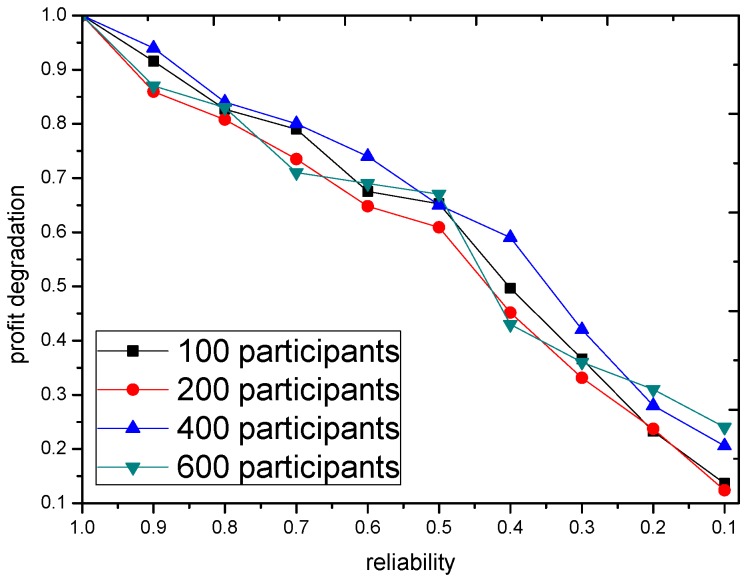
The impact of data reliability to GTSA based on 100 tasks.

However in practice, the reliability of data is important. To further show the impact of data quality to the GTSA, a simple simulation is integrated into our discussion. In this case, some participants uploads their expected trajectory but never follow the trajectory. The participant like this is called the unreliable participant. In this case, the quality of sensing data from the unreliable participants is rather low. The profit degradation by GTSA under different ratio of unreliable participants is shown in Figure 13. The profit degradation is derived by the comparison between profit with unreliable participants and the profit when there is no reliable participants. The result shows that the quality of sensing data influence the performance of GTSA obviously. The more unreliable participants as candidates, the more degradation of performance

Moreover, the trust-based model should be taken into consideration for the reliability and quality of data, because the actual trajectory can be changed, which is common in practice. There are many works to ensure requirements of reliability in vehicular networks, Tang et al. [27] proposes a trustworthy cooperation path generating scheme to ensure the safety of cooperation and increase cooperation completion rates in vehicular cloud computing. In our future works, the more realistic model with sensing quality and trustworthy requirements will be discussed.

Moreover, the configuration of profit weighting factor and the form of the fitness function should be considered, to evaluate the chromosome properly in the new scenario with more realistic requirements.

## 7. Conclusions

This paper studies the scheduling problem of a trading platform in a vehicular participatory sensing network (VPSN). The trading platform in the VPSN tries to maximize the profit by accepting tasks and recruiting participants for accepted tasks. The formal model of VPSN consists tasks, participants, and a trading platform is given. A Genetic-based Trading Scheduling Algorithm (GTSA) is introduced to solve this scheduling problem. The result of simulation based on taxis trajectories in practice shows GTSA is practical and effective for the trading platform to gain the profit in the VPSN.

## Figures and Tables

**Table 1:** The requirements and payments of tasks.

Tasks Γ	Task 1	Task 2	Task 3
**Requirements**	{A,B}	{C}	{B,D}
**Payment**	3	1	2

**Table 2:** The trajectories and bids of participants.

Participants	Participant 1	Participant 2	Participant 3
**Spatial Coverage of Trajectory**	{AC}	{BC}	{CD}
**Bid**	2	1	3

**Table 3:** The different scheduling of trading platform for the motivation.

Selected Tasks	{1}	{2}	{3}	{1,2}	{1,3}	{2,3}	{1,2,3}
**Spatial Coverage Requirements**	AB	C	BD	ABC	ABD	BCD	ABCD
**Payment**	3	1	2	4	5	3	6
**Recruited Participants**	1,2	2	2,3	1,2	1,2,3	2,3	1,2,3
**Cost**	3	1	4	3	6	4	6
**Profit (Payment-Cost)**	0	0	−2	1	−1	−1	0

**Table 4:** The number of useless scheduling by GTSA and POB.

Max Payment of Tasks	100	50	10
**Number of useless scheduling by GTSA**	4	23	183
**Number of useless scheduling by POB**	12	78	239
